# Acknowledgment to Reviewers of *Infectious Disease Reports* in 2021

**DOI:** 10.3390/idr14010012

**Published:** 2022-01-28

**Authors:** Infectious Disease Reports Editorial Office

**Affiliations:** MDPI AG, St. Alban-Anlage 66, 4052 Basel, Switzerland

Rigorous peer-reviews are the basis of high-quality academic publishing. Due to the great efforts of our reviewers, *Infectious Disease Reports* was able to maintain its standards for the high quality of its published papers. Thanks to the contribution of our reviewers, in 2021, the median time to first decision was 19 days and the median time to publication was 41.5 days. The editors would like to extend their gratitude and recognition to the following reviewers for their precious time and dedication, regardless of whether the papers they reviewed were finally published:
A. Robert, PhilippeMa, JianpingAbate, GiuliaMahroum, NaimAbdeltawab, NourtanMalik, HasmatÁbrányi-Balogh, PéterMallela, Shamroop KumarAgoro, RafiouMangiaterra, GianmarcoAhnert, PeterMarcos Tejedor, FélixAlaimo, AlessandroMarín, ClaraAlkan, MichaelMataruna-Dos-Santos, Leonardo JoseAmaro, AnaMatsuda, YuheiAmbegaonkar, AbhijitMattila, SariAnca, ZguraMeans, RobertAnisimov, Andrey P.Meesters, KevinArghittu, AntonellaMenasce, DarioBadmalia, Maulik D.Mentel, GrzegorzBagiu, Iulia-CristinaMiguel, IsabelBarberis, ElettraMikolajczyk, AnitaBartoloni, AlessandroMinutolo, AntonellaBogucki, JacekMitsogiannis, IraklisBoone, Edward L.Moberly, Heather K.Borriello, GiorgiaMoffa, Matthew A.Bosco-Lauth, Angela M.Montalti, MarcoBourgeois, DenisMoretti, SoniaBowman, J. P.Morichau-Beauchant, TristanBraczkowski, RyszardMukundan, SanthoshBrogna, BarbaraMulder, BobBruno Luigi Rocchi, MarcoOhkuri, TakayukiBsisu, IsamOishi, TomohiroBurugupalli, SatvikaOrusa, RiccardoCarneiro, PauloOtsuki, TakemiCasaca, WallaceOyelade, TopeChatterjee, SampurnaPabelick, Christina M.CH-Chaoui, MohamedPark, Jee SooChiu, Chia-YuPatel, PalakCobo, JavierPaul, AlokConti, PioPinto, SandraContreras-Reyes, Javier E.Piubelli, ChiaraCosby, LouisePomara, CristoforoCovrig, Raul-CiprianPouliakis, AbrahamCusick, Matthew F.Pozzi, MauraCuteri, VincezoPuma, PaolaDe Ciuceis, CarolinaQeadan, FaresDeiana, GiovannaQuinto, Emiliano JoséDénes, AttilaRakov, A. V.Dimitrov, KirilRamalingam, SathishkumarDzimianski, JohnRaman, RenukaFalkinham, Joseph O., IIIRaval, YashFanelli, AngelaRawlings, Stephen A.Ferrara, PietroRazai, MohammadFlisiak, RobertRecalcati, SebastianoGharehgozli, OrkidehRoccetti, MarcoGiurg, MirosławRodríguez Villanueva, JavierGodoy-Carter, VeronicaRother, ConnGonzález-Granado, Luis IgnacioRusso, BrianGonzalez-Rodriguez, AlexandreSaldiva, Paulo Hilário NascimentoGouveia, Élvio Rúbio QuintalSánchez De Madariaga, RicardoGrant, MatthewSano, YujiroGroendyke, ChrisScarabello, AlessandraGumerov, Vadim M.Schott, UlfGysi, DeisyScior, ThomasHarazny, Joanna M.Seals, Brenda F.Hayashi, ShyunjiShekhar, RahulHenriksson, JohanShelat, Vishalkumar G.Houhoula, DimitraShibata, YasushiHume, AnnaShokouhimehr, MohammadrezaIanni, AndreaShumilov, EvgeniiIovino, FedericoSimionescu, AncaIrina, ChelyshevaSingal, BhartiJaggavarapu, SiddharthSingh, Amit K.Jankovic, MomciloSingh, Anand PrakashJeffree, Rosalind LindySingh, SukhvinderJohnson, Dylan M.Steinborn, MichaelKaminski, Tomasz W.Steinmeyer, ZaraKamp, ChristelStephens, EdwardKanwar, AnubhavStuchlik, StanislavKato, HideoSwami, UmangKędzierska-Mieszkowska, SabinaTavakoli, Norma P.Keeler, ShamusTeixeira, Miguel CachoKim, Ki-YoungTeo, AndrewKinthada, LakshmanakumarThomas, Pauline A.Kitchen, ChristinaTonelli, MicheleKokkinakis, Emmanouil N.Ulibarri, GerardKoyuncu, Orkide O.Unterweger, ChristineKwun, Hyun JinVrabel, RobertLaabei, MaisemWalker, Louise A.Lacina, LukasWang, WilliamLarrous, FlorenceWölfel, RomanLenhard, Justin R.Xie, ZhiqunLeung, CharZhao, XiangliLeyson, ChristinaZimmermann, Gregor S.Lindoso, José Angelo LaulettaZlatkov Kolev, NikolaLitofsky, Norman ScottZoccoli, AntonioLoncoman, Carlos


